# Immune Challenge Alters Reactivity of Hippocampal Noradrenergic System in Prenatally Stressed Aged Mice

**DOI:** 10.1155/2019/3152129

**Published:** 2019-01-21

**Authors:** Gayane Grigoryan, Niklas Lonnemann, Martin Korte

**Affiliations:** ^1^Division of Cellular Neurobiology, Zoological Institute, Technical University of Braunschweig, Braunschweig 38106, Germany; ^2^Neuroinflammation and Neurodegeneration Group, Helmholtz Centre for Infection Research, Braunschweig 38126, Germany

## Abstract

Prenatal stress (PS) has long-term sequelae for the morphological and functional status of the central nervous system of the progeny. A PS-induced proinflammatory status of the organism may result in an impairment of both hippocampal synaptic plasticity and hippocampus-dependent memory formation in adults. We addressed here the question of how PS-induced alterations in the immune response in young and old mice may contribute to changes in hippocampal function in aging. Immune stimulation (via *LPS injection*) significantly affected the ability of the hippocampal CA3-CA1 synapse of PS mice to undergo long-term potentiation (LTP). Elevated corticosterone level in the blood of aged PS mice that is known to influence LTP magnitude indicates a chronic activation of the HPA axis due to the *in utero* stress exposure. We investigated the contribution of adrenergic receptors to the modulation of hippocampal synaptic plasticity of aged mice and found that impaired LTP in the PS-LPS group was indeed rescued by application of isoproterenol (a nonspecific noradrenergic agonist). Further exploration of the mechanisms of the observed phenomena will add to our understanding of the interaction between PS and proinflammatory immune activation and its contribution to the functional and structural integrity of the aging brain.

## 1. Introduction

In recent years it became evident that early-life adversities have a strong impact on the development of different organ systems, including the nervous and the immune systems [1–7]. It has been shown that prenatal stress (PS) of different intensities, experienced in a certain time window, might have long-term sequelae for the morphological and functional status of the central nervous system (CNS) of the offspring [8–10]. There is a tight correlation between significant alterations in brain morphology [11–13] and impaired synaptic plasticity in the hippocampus and frontal cortex of the PS prepubertal as well as adult animals [14–19]. Exposure to stress during foetal life may cause as well changes in the emotional status of the progeny, putting them at risk to develop anxiety-related and other psychiatric diseases, including schizophrenia [2, 20–25]. Moreover, PS-induced changes are expressed in an age- and sex-dependent manner and all together may underlie altered abilities to learn and form new memories [17, 26].

One of the important neuromodulators that is involved in mediating age-dependent changes in synaptic plasticity and is associated with the stress response is norepinephrine (NE) [27–34]. It has been shown that the concentration of NE in the cerebral cortex and locus coeruleus is significantly reduced in PS rats. In addition, the concentration of NE metabolites was significantly elevated in PS rats, suggesting an increased turnover of brain NE [34]. In our own study, we observed differential modulation of LTP mediated by activation of *β*1-adrenergic receptors in the hippocampus of young PS rats [35, 36].

It is believed that the negative effect of PS is mediated by chronic activation of the hypothalamic-pituitary-adrenal axis (HPA) [37, 38] that plays a key role in the reaction to immune stimuli. It is noteworthy that PS induces a proinflammatory status of the organism [7, 39], which in turn may potentiate neuroinflammatory responses to peripheral and central immune challenges in the later life [40–46]. At the same time, there is a growing evidence that increased expression of proinflammatory cytokines impairs both hippocampal synaptic plasticity [47–51] and hippocampus-dependent memory formation in adult rodents [52–56].

Therefore, in the present study we addressed the question of how PS induced alterations in the reactivity of HPA axis and of how the immune response in the offspring may contribute to alterations in hippocampal function during aging. Because aged individuals (in comparison to young adults) are more likely to suffer memory impairments following a traumatic life event, including an infection, we compared the effects of an immune challenge (sterile infection model [57]) in both young (one-month-old) and old (eight-month-old) naïve and PS mice.

## 2. Materials and Method

One-year-old C57BL6/J mice of both genders were used in the study. All experimental procedures had been conducted in accordance with the European and National regulations and approved by the responsible authorities (33.9-42502-04-14/1559).

### 2.1. Prenatal Stress Induction

Pregnant C57BL6/J mouse dams were stress exposed at the last week of gestation (from day E14 to day E21) that coincides with the time window of embryonic neurogenesis of hippocampal CA1 area (day 14 and with peak on day 15) [58, 59]. Therefore, it is considered to be the vulnerable time period for the development of hippocampal formation. Prenatal stress (PS) was induced by exposing pregnant mice to two consecutive sessions of nonpredictable stress factors. The first treatment (gestation days 14 and 18) includes a swim in a water bucket (of room temperature) for 15 min (forced swim stress). The second treatment (gestation days 15 and 19) includes a restraint in a transparent tube for 45 min (restraint stress). The tube is narrow with two small holes on the poles (one for the nose to allow easy breathing and second for the tail fixation, so that the mouse cannot change its body position). The third treatment (gestation days 16 and 20) includes placement on an elevated platform for 30 minutes. Placement on an elevated platform has been shown to lead to an increase of both plasma corticosterone and hippocampal acetylcholine efflux [59, 60]. The described stress protocol has been adapted from Yaka et al. [17], who found a 10-fold increase of maternal plasma corticosterone level after forced swim or restraint, and has been used by us before [23, 35]. Control pregnant dams were left undisturbed.

### 2.2. Immune Activation

To challenge the peripheral innate immunity in young and old (one- and eight-month-old, correspondingly) naïve and PS offspring of both genders, mice received an intraperitoneal injection (i.p.) of bacterial endotoxin (lipopolysaccharide (LPS)) of *Salmonella enterica serogroup Typhimurium* (*S. typh.* (Sigma-Aldrich L 6511), freshly prepared from a stock solution by dilution in 0.9% NaCl; two consecutive days, 0.2 *μ*g/g body weight (B.W.)). Mice were habituated to daily handling, and body weight measures were taken for at least 3 days prior to and for at least 1 week after immune stimulation.

Naïve and PS mice that received an injection of sterile NaCl (0.9%, at the volume calculated at 1 *μ*l/g B.W.) served as a control group. They were exposed to the same daily handling procedures.

### 2.3. Electrophysiology

We examined the processes of synaptic plasticity of CA3-CA1 synapse in the hippocampus by recording evoked field excitatory postsynaptic potentials (fEPSPs) from the *stratum radiatum* of acute hippocampal slices taken from one-year-old mice. Mice were anesthetized with high concentration of CO_2_ and decapitated. The brain was removed from the skull into carbogenated (5% CO_2_ and 95% O_2_) ice-cold artificial cerebrospinal fluid (ACSF, containing (in mM) 124 NaCl, 4.9 KCl, 1.2 KH_2_PO_4_, 2.0 MgSO_4_, 2.0 CaCl_2_, 24.6 NaHCO_3_, 10 glucose, at pH = 7.4) and split into two parts. One hemisphere was then transferred into Golgi-Cox staining solution to allow further morphological analysis (for details, see the corresponding section below). The second hemisphere was processed as follows. The hippocampus was isolated from surrounding tissues, and transverse hippocampal slices of 400 *μ*m were cut by using the tissue chopper. The collected dorsal hippocampal slices were transferred into the interface brain slice chamber (Scientific System Design) and incubated at 32°C for at least 1.5 h in the presence of the constant flow of carbogenated ACSF (flow rate of 1 ml/min). A set of two monopolar stimulating and one recording stainless-steel electrodes (5 MΩ; AM-Systems) was placed in the slice. Stimulating electrodes placed on both sides and equidistant from the recording electrode were used to stimulate the Schaffer collaterals and evoke synaptic responses recorded in the *stratum radiatum* of the CA1 area. Therefore, two independent stimulation channels were used for each slice. An input-output curve (dependence of fEPSP slope on stimulation intensity) was plotted prior to each experiment. The stimulation intensity was adjusted to evoke 40% of maximum fEPSP slope and kept constant during the entire recording session.

Alterations of short-term synaptic plasticity were examined by delivering a pair of pulses of the same intensity (paired-pulse protocol). We varied interpulse intervals (IPIs, at 10, 20, 40, 60, 80, and 100 ms) and monitored changes of the second (test) EPSP in regard to the first (conditioning) EPSP (paired-pulse facilitation or depression). To induce long-term synaptic plasticity, theta burst stimulation (TBS, 10 trains of 4 pulses at 100 Hz with an interburst interval of 200 ms, applied 3 times in 10 s intervals) was delivered after 20 minutes of baseline recordings. TBS stimulation of one pathway did not cause any significant change in response to stimulation of the second pathway, indicating their independence. To examine modulatory effect of activation of the noradrenergic system on synaptic activity, a second TBS was delivered to the previously unpotentiated pathway after 15 minutes of application of isoproterenol (Iso, 1 *μ*M, Sigma-Aldrich, dissolved from a stock solution into the recording ACSF immediately before use), a nonselective *β*-adrenergic agonist.

Data acquisition and off-line analysis were performed using Intracell software. The fEPSP slope elicited by stimulation of the Schaffer collaterals was measured over time, normalized to baseline, and plotted as a mean ± SEM.

### 2.4. Corticosterone Measurement

Following the decapitation of the mice for electrophysiological examination, the trunk blood was collected. The blood serum was extracted via centrifugation (3500 r.p.m. for 10 min at 4°C) and used for measurement of corticosterone (CORT) concentrations using the Corticosterone ELISA kit RE52211 (IBL International, Hamburg, Germany) according to the manufacturer's instructions.

### 2.5. Dendritic Spine Density Analysis

In order to analyze the possible alterations in spine density caused by PS and immune challenge (or their combination), we performed Golgi-Cox staining of one brain hemisphere. The staining was performed using the Golgi staining kit (FD NeuroTechnologies) according to the manufacturer's instructions. Briefly, one brain hemisphere was immersed into 2 ml of a mixture of kit solutions A and B (1 : 1) that was exchanged after 24 h and stored at room temperature for 2 weeks to allow complete penetration of the liquid. Then, the brain hemisphere was transferred into solution C that was also renewed after the first 24 h. Brain tissue was kept in a solution C for at least 48 h and up to 7 days before sectioning. All procedures were performed under dark conditions.

Brain coronal sections of 200 *μ*m were cut using a vibrating microtome (Leica, VT1200S) while embedded in 2% agar in 0.1 M PBS. Each section was mounted with solution C on an adhesive microscope slide precoated with 1% gelatin/0.1% chromalaun on both slides and stained according to the manufacturer's protocol with the exception that AppliClear (AppliChem) was used instead of xylene. Stained slices were cover slipped with PermountTM (Thermo Fisher Scientific) and allowed to dry for several days prior to imaging.

Imaging of apical dendritic branches of hippocampal CA1 pyramidal neurons was done with an Axioplan 2 imaging microscope (ZEISS) using a 63 oil objective (NA 1.3) and a z-stack thickness of 0.5 mm under reflected light. The number of spines was determined per micrometer of dendritic length using ImageJ (1.48v, National Instruments of Health, USA). Three to four animals per group and 9–10 neurons per animal were analyzed. Spine density is expressed as a mean ± SEM.

### 2.6. Data Analysis

All numerical data are expressed as a mean ± SEM. Field EPSP slope changes after TBS stimulation were calculated with respect to baseline. Statistical evaluation for electrophysiological experiments as well as for morphological analysis was performed by one-way ANOVA that was followed by Tukey tests. *p* values of <0.05 were considered a significant difference between means.

## 3. Results

### 3.1. Long-Term Effects of an Immune Challenge at One Month of Age

**Figure 1:**
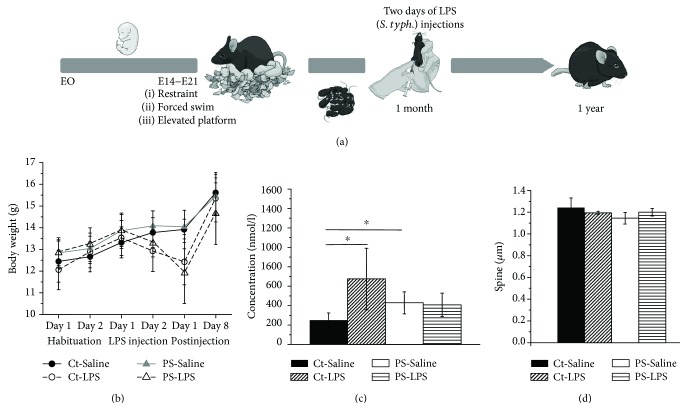
(a) An experimental timeline indicating time windows for an exposure to prenatal stress (PS, at E14–E21) as well as the immune challenge by an injection of lipopolysaccharide from *Salmonella Typhimurium* (LPS, *S. typh.*, 0.2 *μ*g/g B.W., two consecutive days, at one month of age). All mice were examined at the age of one year to perform electrophysiological, morphological, and biochemical analyses of the brain tissue and blood serum. (b) Curves showing body weight alterations prior to, during, and after injections (saline or LPS) for all groups. Mice that received vehicle treatment showed no alteration in their body weights. Body weights taken one week after the beginning of immune stimulation showed full recovery of an initial drop that was induced by immune challenge. Note that PS-LPS mice were smaller than their littermates treated with saline. (c) A bar graph demonstrating corticosterone plasma levels in all experimental groups. (d) Spine analysis of Golgi-Cox-stained brain sections did not reveal significant changes in the spine density of apical dendrites of hippocampal CA1 pyramidal neurons between different treatment groups. Note that spine density of hippocampal CA1 principal cells was affected in Ct-LPS and PS-Saline mice. All values are expressed as means ± SEMs (^∗^*p* < 0.05).

We examined the long-term impact of PS and its combination with an immune activation in juvenility on the aging mouse brain (schematic of experimental timeline in Figure 1(a)). Naïve and prenatally stressed (PS) C57BL6/J male and female mice were immune challenged by two-day consecutive i.p. injections of lipopolysaccharide from *Salmonella Typhimurium* (LPS, *S. typh.*, 0.2 *μ*g/g B.W.) at the age of one month. The control group received a vehicle treatment (saline, 0.9% NaCl, 1 *μ*l/g B.W.). This design yielded in the following experimental groups: naïve mice treated either with saline (Ct-Saline) or LPS of *S. typh*. (Ct-LPS) and PS mice treated either with saline (PS-Saline) or LPS of *S. typh*. (PS-LPS).

We monitored the behavioral and body weight alterations induced by the two-day immune stimulation. The immune challenge resulted in a typical sickness behavior with a decrease in motility as well as in body weight (maximal drop on the 3rd day after beginning of the treatment) due to anorexic effects of the LPS challenge [61]. Mice that received vehicle treatment showed no alteration in behavior or body weight. One week after the beginning of immune stimulation, all animals showed full recovery from the procedure. Mice from all groups gained weight since they were all in their live growing phase. However, PS-LPS mice lost more weight than their littermates treated with saline, indicating that PS mice recovered slower after immune stimulation (Figure 1(b)).

Animals of all four groups were examined at the age of one year. Morphological, electrophysiological, and biochemical analysis of the brain tissue and blood serum was performed. We observed a two-fold increase in corticosterone (CORT) levels in the blood of PS-Saline mice (428.52 ± 112.59 nmol/l (*n* = 6, *p* < 0.05, *F* value 1.39) compared with 247.05 ± 77.86 nmol/l (*n* = 4) in the Ct-Saline group), which testifies to the chronic activation of the HPA axis due to *in utero* stress exposure. LPS treatment led to an elevation of CORT level in naïve mice (672.58 ± 316.78 nmol/l (*n* = 4, *p* < 0.05, *F* value 1.7), in the Ct-LPS group). Interestingly, immune activation of PS mice did not induce a further increase in CORT level (407.4 ± 119.73 nmol/l (*n* = 6), not significant when compared with PS-Saline mice) (Figure 1(c)).

Analysis of Golgi-Cox-stained apical dendrites of CA1 pyramidal neurons did not reveal significant changes in the spine density between different treatment groups. However, we observed some decrease of spine density of hippocampal CA1 principal cells in Ct-LPS and PS-Saline mice (Figure 1(d)).

**Figure 2:**
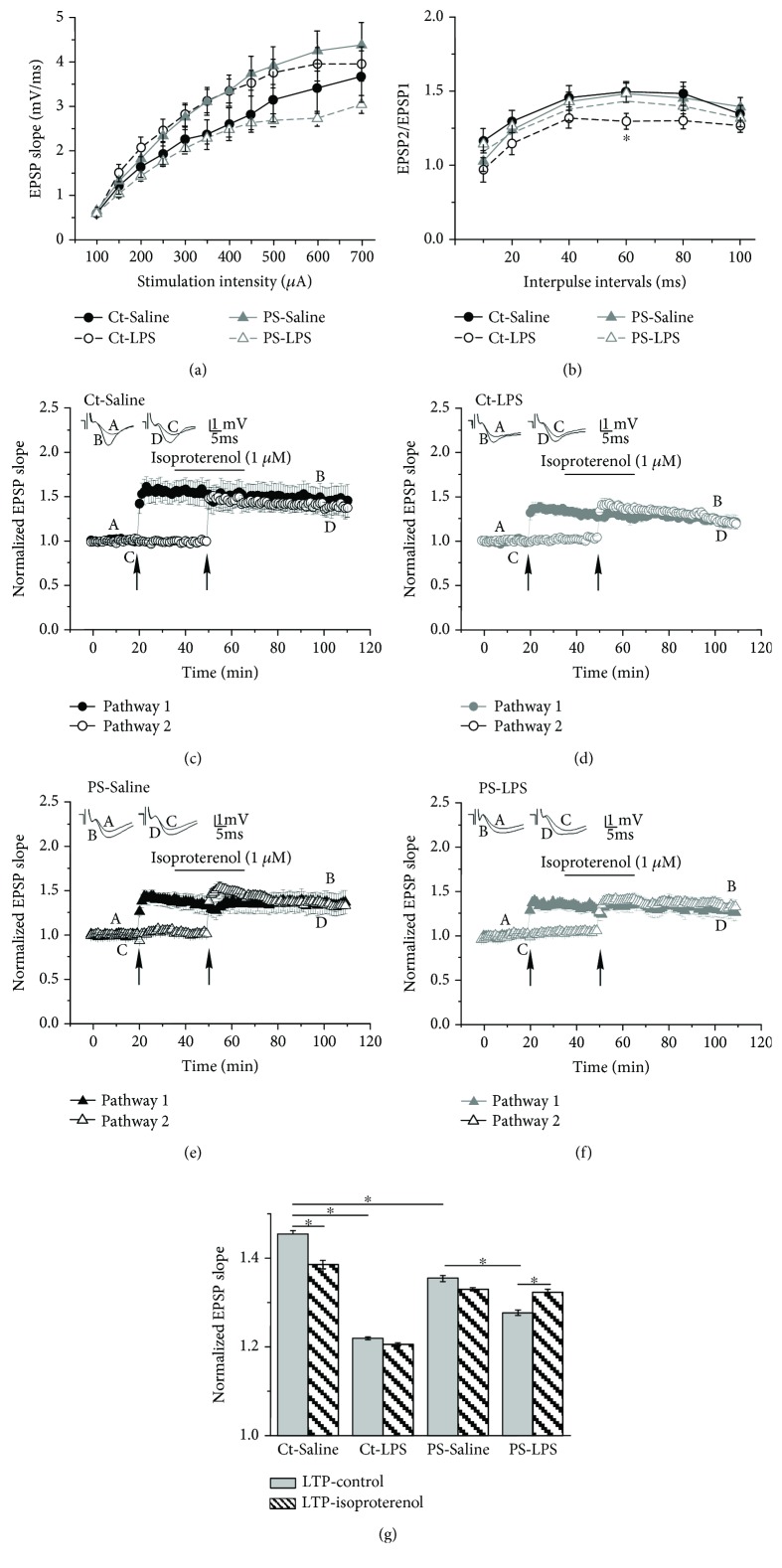
LPS treatment in juvenility (at one month of age) induces long-lasting changes of long-term hippocampal plasticity in both control (Ct) and prenatally stressed (PS) mice, including alterations in the reactivity to the activation of the adrenergic system. (a) Input-output curves of excitatory postsynaptic potential (EPSP) slopes for dorsal hippocampal slices of control (Ct) and prenatally stressed (PS) mice showed no significant difference between saline and LPS treatments. (b) Examination of paired-pulse facilitation (PPF) of the EPSP slopes expressed as a response to the 2nd stimulation over the 1st at different interpulse intervals (IPIs; 10, 20, 40, 60, 80, and 100 ms) in Ct and PS slices. No difference in PPF was found between saline and immune challenged slices, except for a significant decrease of PPF in the Ct-LPS group at 60 ms interval. (c–f) Effect of isoproterenol (Iso, 1 *μ*M), a nonselective *β*-adrenergic agonist, application on long-term potentiation (LTP) of CA3-CA1 synapse activity recorded from the *stratum radiatum* of dorsal hippocampal slices. LTP was induced by application of theta burst stimulation (TBS, 10 trains of 4 pulses at 100 Hz with an interburst interval of 200 ms, applied 3 times in 10 s intervals). The first TBS was delivered to pathway 1 after 20 minutes of baseline recording. The second TBS was applied to the previously unpotentiated pathway 2 after 15 minutes of washing in Iso (the bar shown above the record). The arrows denote the points at which TBS was delivered. Insets in (c–f) are sample illustrations of the EPSPs recorded at the times indicated in the averaged traces (a–d) before and after the TBS application. Hippocampal slices from PS mice showed an impaired ability to undergo LTP in response to TBS. LPS injections significantly affected LTP magnitude in aged animals of both Ct-LPS and PS-LPS groups. The facilitating effect of Iso on LTP was observed in the PS-LPS group only. (g) Summary bar graph of the results shown in (c–f). ^∗^Significant differences at *p* < 0.05.

Short- and long-term plasticity of CA3-CA1 synapses was investigated by electrophysiological examination of evoked fEPSPs recorded in *stratum radiatum* in response to stimulation of Schaffer collaterals in the CA1 area in acute slice preparations taken from one-year-old mice. First, we looked at possible PS- or LPS-induced alterations in basal synaptic transmission by plotting an input-output curve that shows the dependence of the EPSP slope on stimulation intensity. We observed no significant alterations in basal synaptic transmission between all groups of animals (Figure 2(a)). In order to investigate changes in short-term synaptic plasticity, we applied a paired-pulse protocol and analyzed how the second (test) EPSP slope changed in regard to the first (conditioning) one dependent on interpulse intervals (IPIs). We found a reduction of paired-pulse facilitation in the Ct-LPS group compared to Ct-Saline littermates (*p* < 0.05, *F* value 5.09, at IPIs of 60 ms). Paired-pulse facilitation in LPS-treated PS mice did not differ from values in the PS-Saline group (Figure 2(b)).

To examine long-term synaptic plasticity and its modulation by an activation of the noradrenergic system, we applied theta burst stimulation (TBS) to both recording pathways. The first TBS was delivered to pathway 1 after 20 minutes of baseline recording and evoked long-term potentiation (LTP) of synaptic activity of the CA3-CA1 connection. The second TBS was applied to the previously unpotentiated pathway 2 after 15 minutes of washing in isoproterenol, a nonselective *β*-adrenergic agonist (Iso, 1 *μ*M) (Figures 2(c)–2(f)). We found that CA3-CA1 synapses in the hippocampus of PS mice have a significantly impaired ability to undergo LTP in response to TBS. LTP magnitude in the PS-Saline group was reduced by ~10% (1.35 ± 0.01 (*n* = 12), *p* < 0.05, *F* value 5.57) in comparison with 1.45 ± 0.01 (*n* = 10 in the Ct-Saline group). Injection of an infectious agent in juvenility significantly affected LTP magnitude in one-year-aged animals (1.21 ± 0.002 (*p* < 0.001, *F* value 5.38, *n* = 10) in the Ct-LPS group when compared with the Ct-Saline group). In PS mice LPS treatment also led to a decrease in magnitude of LTP of ~9% (1.27 ± 0.01, *n* = 14, *p* < 0.05, *F* value 6.45) in the PS-LPS group compared with the PS-Saline group) (Figures 2(c)–2(g)).

Many studies implicated the adrenergic system of the brain as being involved in the mediation of both stress and neuroinflammatory responses [27–32, 62]. Moreover, it undergoes age-dependent changes that are believed to contribute to the development of age-related brain dysfunctions [33, 63, 64]. We investigated how activation of adrenergic receptors by application of isoproterenol (Iso, 1 *μ*M) modulates hippocampal LTP expression in aged PS- and immune-stimulated mice. We found that in slices from aged PS-Saline as well as from Ct-LPS mice, 15 min of application of Iso failed to facilitate TBS-induced LTP (Figures 2(d), 2(e), and 2(g)). Interestingly, there was a decrease in LTP magnitude in Ct-Saline slices (to 1.38 ± 0.01, *n* = 10, *p* < 0.05, *F* value 8.86) (Figures 2(c) and 2(g)). Most interestingly, the facilitating effect of Iso on LTP magnitude was observed in the PS-LPS group only (1.32 ± 0.01, *n* = 14, *p* < 0.05, *F* value 14.99) (Figures 2(f) and 2(g)).

In conclusion, we could show with these experiments that LPS treatment in juvenility induces long-lasting impairments in long-term hippocampal plasticity in both Ct and PS mice, including alterations in the reactivity to the activation of the adrenergic system.

### 3.2. Immune Challenge of Eight-Month-Old Mice Affects Synaptic Plasticity in the Hippocampus

**Figure 3:**
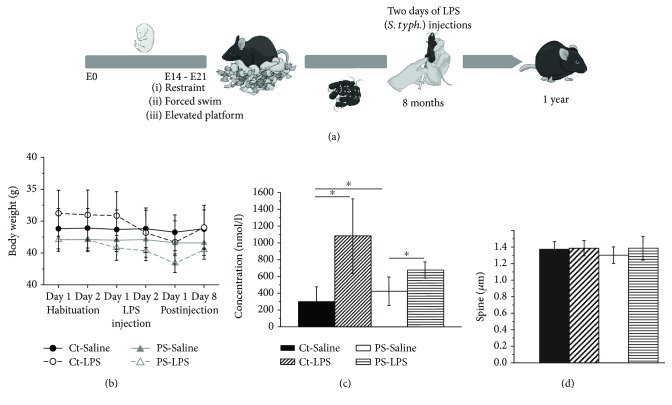
(a) An experimental timeline indicating time windows for an exposure to prenatal stress (PS, at E14–E21) as well as the immune challenge by an injection of lipopolysaccharide from *Salmonella Typhimurium* (LPS, *S. typh.*, 0.2 *μ*g/g B.W., two consecutive days, at eight month of age). All mice were sacrificed at the age of one year to perform electrophysiological, morphological, and biochemical analyses of the brain tissue and blood serum of aging animals. (b) Curves showing body weight alterations prior to, during, and after saline and LPS injections for all groups. Note that PS mice were smaller than control ones, in general. All mice that were injected with an LPS showed body weight drop. All mice recovered and body weight values reached the level of saline-treated littermates. (c) A bar graph demonstrating elevation of corticosterone plasma levels following exposure to PS and LPS injections. (d) Dendritic spine analysis of Golgi-Cox-stained brain sections revealed no alterations in the spine density of hippocampal CA1 pyramidal neurons between different treatment groups. All values are expressed as means ± SEMs (^∗^*p* < 0.05).

A similar experimental design was used to study the consequences of immune challenge in old (eight months of age) animals (Figure 3(a)). We monitored behavior and body weight of the animals during the habituation as well as during the immune stimulation and postinjection sessions. Experimental manipulations did not cause a change in body weight in both the Ct-Saline and the PS-Saline groups. Note that PS mice were smaller than control animals, in general. All LPS-injected mice showed a body weight drop which was most pronounced on the third day after beginning of the treatment. All mice recovered and body weight values reached the level of saline-treated littermates one week postinjection (Figure 3(b)).

Examination of blood CORT level revealed significantly elevated values in all groups (1083.07 ± 445.30 nmol/l (*n* = 3, *p* < 0.05, *F* value 1.003) in the Ct-LPS group and 423.87 ± 167.36 (*n* = 3, *p* < 0.05, *F* value 0.9) in the PS-Saline group, compared with 297.37 ± 180.15 nmol/l (*n* = 3) in the Ct-Saline group). LPS challenge of PS mice led to a significant increase of CORT level in PS-LPS animals (676.37 ± 96.19 nmol/l, *n* = 3, *p* < 0.05, *F* value 0.64, compared with PS-Saline animals) (Figure 3(c)).

The dendritic spine density investigated at the age of one year and two months after immune challenge was not altered in both Ct-LPS and PS-LPS groups (Figure 3(d)).

**Figure 4:**
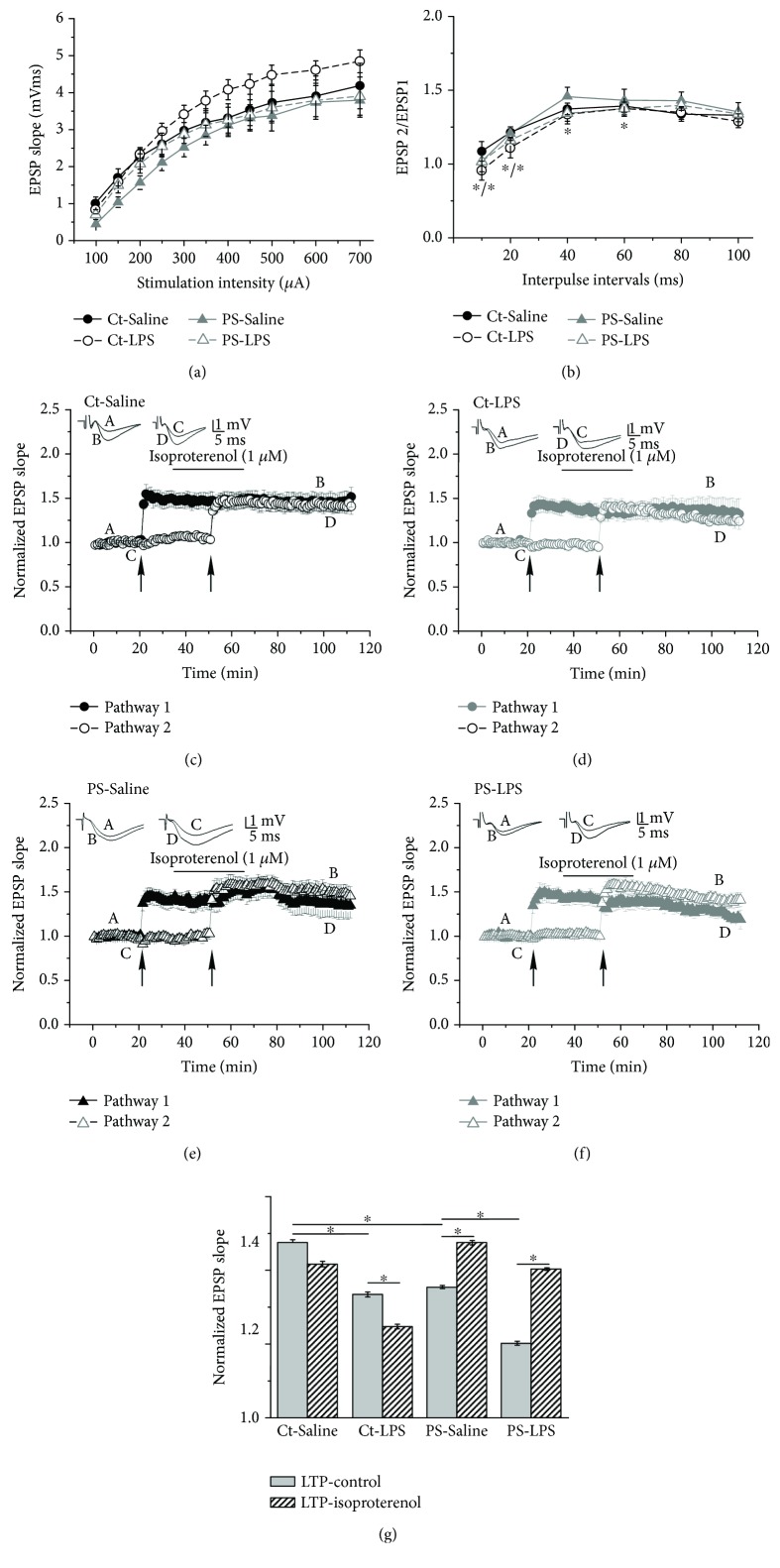
LPS treatment of eight-month-old mice had a profound effect on LTP in prenatally stressed mice. (a) Input-output curves of excitatory postsynaptic potential (EPSP) slopes for dorsal hippocampal slices of control (Ct) and prenatally stressed (PS) mice showed no significant difference between saline and LPS treatment groups. Note that LPS treatment led to some increase in basal synaptic transmission in Ct, but not in the PS group. (b) Examination of paired-pulse facilitation (PPF) of the EPSP slopes expressed as a response to the 2nd stimulation over the 1st at different interpulse intervals (IPIs; 10, 20, 40, 60, 80, and 100 ms) in Ct and PS slices. LPS treatment led to a significant decrease in paired-pulse facilitation at 10 ms and 20 ms of IPIs in the Ct-LPS group as well as at IPIs of 20 ms, 40 ms, and 60 ms in the PS-LPS group. (c–f) Effect of isoproterenol (Iso, 1 *μ*M), a nonselective *β*-adrenergic agonist, application on long-term potentiation (LTP) of CA3-CA1 synapse activity recorded from *stratum radiatum* of dorsal hippocampal slices. LTP was induced by application of theta burst stimulation (TBS, 10 trains of 4 pulses at 100 Hz with an interburst interval of 200 ms, applied 3 times in 10 s intervals). The first TBS was delivered to pathway 1 after 20 minutes of baseline recording. The second TBS was applied to the previously unpotentiated pathway 2 after 15 minutes of washing in Iso (the bar shown above the record). The arrows denote the points at which TBS was delivered. Insets in (c–f) are sample illustrations of the EPSPs recorded at the times indicated in the averaged traces (a–d) before and after the TBS application. Immune challenge at eight months of age led to a significant impairment of LTP magnitude in both Ct and PS groups. Activation of the adrenergic system by application of Iso did not alter LTP expression in Ct-Saline and Ct-LPS groups but was instrumental in facilitating LTP in both PS-Saline and PS-LPS groups. (g) Summary bar graph of the results shown in (c–f). ^∗^Significant differences at *p* < 0.05.

Examination of basal synaptic transmission of hippocampal slices taken from one-year-old mice that experienced immune stimulation at eight months of age showed no difference between the Ct and PS groups. However, LPS treatment led to an increase in basal synaptic transmission in the Ct group (seen as a left shift of the input-output curve) but no change in the PS group (Figure 4(a)). As to short-term plasticity, LPS treatment led to a significant decrease in paired-pulse facilitation at IPIs of 10 ms (*p* < 0.05, *F* value 1.6) and 20 ms (*p* < 0.05, *F* value 2.65) in the Ct-LPS group as well as in the PS-LPS group at IPIs of 20 ms (*p* < 0.05, *F* value 0.21), 40 ms (*p* < 0.05, *F* value 0.31), and 60 ms (*p* < 0.05, *F* value 0.15) (Figure 4(b)). We investigated long-term synaptic plasticity in all treatment groups and found a significant impairment in potentiation efficacy of the hippocampal CA3-CA1 synapse in the Ct group as well as in the PS-LPS group caused by an immune challenge at eight months of age. There was a significant decrease in LTP magnitude caused by LPS treatment in both Ct-LPS slices (1.33 ± 0.01 (*n* = 11, *p* < 0.05, *F* value 2.13) compared with 1.47 ± 0.01 (*n* = 11) in the Ct-Saline group) and PS-LPS slices (1.2 ± 0.01 (*n* = 11, *p* < 0.05, *F* value 2.48) compared to 1.35 ± 0.004 (*n* = 7, *p* < 0.05, *F* value 2.86 (compared to the Ct-Saline group)) in the PS-Saline group).

Application of Iso (1 *μ*M) and activation of the adrenergic system did not alter LTP expression in Ct-Saline and Ct-LPS groups (1.42 ± 0.01 and 1.24 ± 0.01, respectively) but was instrumental in facilitating LTP in PS-Saline and PS-LPS groups (1.47 ± 0.01 (*p* < 0.05, *F* value 2.07) and 1.40 ± 0.004 (*p* < 0.05, *F* value 5.36), respectively) (Figures 4(c)–4(g)).

## 4. Discussion

In our study we investigated the question of how the exposure to traumatic events during the *in utero* development primes neuroinflammatory responses in young and old mice leading to remodelling of hippocampal synaptic plasticity in aged animals. It is known that consequences of stress, in particular of PS, might be long term and critically depend on the time window of influence and intensity of stress factors (for review, see [9, 10]). The “mild” stress protocol utilized by Yaka et al. [17], adapted by us earlier [23, 35], was used in the present study. The sequence of unpredictable different stressors such as restraint, forced swim, and an exposure to an open space was applied to pregnant mice in order to prevent the possible adaptation to stressors. Stress-mediated activation of the maternal HPA axis resulting in an elevation of the concentration of corticosterone in the maternal blood [17, 65] causes changes in the development of sensitive tissues of the foetus, in particular, of catecholaminergic neurons [66]. This is considered to be critical for a stress-triggered pathology. In the present study we observed significantly increased values of basal corticosterone levels in the blood plasma of aged (one-year-old) PS mice, which indicates to a chronic activation of the HPA axis of the PS offspring. This finding correlates to and extends the results of others that observed prolonged and increased corticosterone secretion in response to external stimuli due to altered reactivity of the HPA axis and functional changes of different types of corticosteroid receptors in stressed animals [37, 38, 67–71]. Glucocorticoids are considered as anti-inflammatory hormones that suppress inflammation and other immunologically mediated processes [72]. However, there is evidence that under some conditions glucocorticoids can sensitize central proinflammatory responses [44, 73]. In our current study, we observed a significant increase in basal corticosterone levels in naïve aged mice that were immune challenged in juvenility. Interestingly, aged LPS-treated PS mice exhibited a decrease in basal corticosterone values suggesting a reduced efficacy of the corticosterone feedback mechanism in PS animals [37].

Several studies addressed the question of PS-associated changes in learning behavior and underlying processes of synaptic plasticity in particular in the hippocampus [14–19, 74]. However, in most of those studies, the authors investigated functional as well as structural alterations in juvenile or young-adult PS rats and mice. In the present study, we examined hippocampal synaptic plasticity in middle-aged (one-year-old) mice, when senescent changes can already be detected [75]. We were able to show that *in utero* stress exposure had a long-term profound impact on synaptic plasticity as we found a significant impairment in the ability of CA3-CA1 hippocampal synapses to undergo facilitation in aging PS mice. LPS challenge in juvenility as well as at eight months of age affected LTP magnitude in both naïve and PS animals. However, we found that old PS mice appeared to be more vulnerable and exhibited higher susceptibility to LPS treatment and stronger decline in LTP magnitude than mice from the control group. The impairment of hippocampal synaptic plasticity in PS mice could be explained by stress-induced malfunction of molecules/systems that are involved in LTP. Thus, the downregulation of the tissue plasminogen activator was observed in PS animals. This results in turn in an imbalance in levels of the mature brain-derived neurotrophic factor (BDNF) that has been shown to be crucial for LTP induction and maintenance [5, 18, 26, 65, 76, 77]. The reduction in NR1 and NR2B subunits of the NMDA receptors as well as changes in their functional activity and distribution caused by PS exposure might represent another mechanism involved [13, 14, 17, 78]. The short-term plasticity, examined by the paired-pulse ratio, did not show significant changes in Ct and PS mice that were LPS treated at one month of age. However, a LPS challenge at eight months of age led to a decrease of the paired-pulse ratio at short intervals both in Ct and PS mice. We can speculate that observed alteration in a paired-pulse ratio is most likely dependent on the age- and treatment-dependent decrease of a residual Ca^2+^ level in the presynaptic terminal caused by a first conditioning stimulus.

The noradrenergic neuromodulatory system plays an important role in the stress response and can enhance synaptic plasticity [35, 36, 30]. In our own study we were able to show that PS selectively suppresses noradrenergic effects of conversion of short-term potentiation into long-term potentiation in the dorsal hippocampus of young rats [35]. In this study, we did not find facilitation of LTP in the dorsal hippocampus in response to activation of the NE system by application of isoproterenol in Ct-Saline mice. Moreover, there was a trend of a decrease in LTP magnitude upon the presence of isoproterenol. We believe that the observed lack of facilitation of LTP in the dorsal hippocampus has an age-dependent character. We hypothesize that it is mediated by PS-induced reduction of concentration of NE [34] and possible degenerative alteration of noradrenergic neurons and fibres that occur in old age [79, 80]. It is important to note that the atrophy and loss of noradrenergic cells in the locus coeruleus is also observed in neurodegenerative diseases. In Alzheimer's disease, it correlates with the degree of deposition of amyloid plaques and neurofibrillary tangles as well as with the severity of cognitive deficits both in mouse models and in human Alzheimer's patients [79, 81, 82]. Interestingly, we observed significant isoproterenol-mediated facilitation of LTP in the PS-Saline group that was handled at eight months, but not at one month of age. This facilitation was strong and led to an increase of the LTP magnitude to the level in the control group. We found a different reaction of slices from Ct-LPS and PS-LPS middle-aged mice with regard to application of isoproterenol and therefore to the activation of the NE system as well as with regard to two different age-treatment (at one and at eight months) groups. Thus, isoproterenol application to slices from the Ct-LPS group that was immune challenged in juvenility had no effect on LTP magnitude, while in slices from mice challenged at eight months, it resulted in a significantly decreased LTP. In striking contrast to these observations, there was a significant facilitation of LTP in response to activation of NE receptors in both PS-LPS groups, however, with a stronger effect in slices from mice that were immune challenged at eight months. One of the possible mechanisms that could be involved in the restoration of LTP in the LPS-treated group upon NE activation is the known function of norepinephrine as an endogenous anti-inflammatory factor capable of modulating the expression of inflammatory cytokines, including IL-1*β* and IL-6 [83–86]. The exaggerated elevation of inflammatory cytokines and an increase in the number of activated microglial cells that are reported to occur in response to both stress and immune activation may lead to impairments in synaptic plasticity and spatial memory in aged animals [55, 87–89]. The involvement of epigenetic alterations in a NE-mediated modulation of synaptic plasticity upon PS also has to be considered [6]. The further exploration of the mechanisms of the observed phenomena will add to our understanding of cross-sensitizing of PS and proinflammatory immune activation.

## 5. Conclusions

LPS treatment of eight-month-old mice has a significantly stronger effect on synaptic plasticity, on LTP, in particular, in PS mice. Most notably, impaired LTP magnitude in the PS-LPS group was rescued by application of Iso. This Iso-mediated facilitation of LTP was stronger than the one observed in slices from PS mice treated with LPS at one month of age. Further examination of the mechanisms of the observed phenomena is important for our understanding of how early life experiences may shape the responses to late life challenges and therefore contribute to the functional and structural integrity of the aging brain.

## Data Availability

The data used to support the findings of this study are available from the corresponding author upon request.
